# Donanemab: Appropriate use recommendations

**DOI:** 10.1016/j.tjpad.2025.100150

**Published:** 2025-03-27

**Authors:** G.D. Rabinovici, D.J. Selkoe, S.E. Schindler, P. Aisen, L.G. Apostolova, A. Atri, S.M. Greenberg, S.B. Hendrix, R.C. Petersen, M. Weiner, S. Salloway, J. Cummings

**Affiliations:** a Memory & Aging Center, Departments of Neurology, Radiology and Biomedical Imaging, University of California San Francisco, San Francisco, CA, USA; b Ann Romney Center for Neurologic Diseases, Department of Neurology, Brigham and Women’s Hospital, Harvard Medical School, Boston, MA, USA; c Department of Neurology, Washington University School of Medicine, St. Louis, MO, USA; d Alzheimer’s Treatment Research Institute, University of Southern California, San Diego, CA, USA; e Departments of Neurology, Radiology, Medical and Molecular Genetics, Indiana University School of Medicine, Indianapolis, Indiana, USA; f Banner Sun Health Research Institute, Banner Health, Sun City, AZ, USA; g Center for Brain/Mind Medicine, Department of Neurology, Brigham and Women’s Hospital, Harvard Medical School, Boston, MA, USA; h Department of Neurology, Massachusetts General Hospital, Harvard Medical School, Boston, MA, USA; i Pentara Corporation, Millcreek UT, USA; j Department of Neurology, Mayo Clinic, Rochester, MN, USA; k Departments of Radiology and Biomedical Imaging, Medicine, Psychiatry and Neurology, University of California San Francisco, San Francisco, CA, USA; l Butler Hospital and Warren Alpert Medical School of Brown University, Providence RI, USA; m Chambers-Grundy Center for Transformative Neuroscience, Department of Brain Health, Kirk Kerkorian School of Medicine, University of Nevada Las Vegas, Las Vegas, NV, USA

**Keywords:** Donanemab, Appropriate use recommendations, Amyloid-targeting therapies, Antiamyloid monoclonal antibodies, Expert guidelines

## Abstract

Donanemab (Kisunla^®^), an IgG1 monoclonal antibody targeting N-terminal pyroglutamate-modified forms of amyloid-*β*, is approved in the United States for treatment of early symptomatic Alzheimer’s disease (AD). Appropriate Use Recommendations (AUR) were developed to guide the implementation of donanemab in real-world practice, prioritizing safety considerations and opportunity for effectiveness. The AUR were developed by the AD and Related Disorders Therapeutic Workgroup by consensus, integrating available data and expert opinion. Appropriate candidates for donanemab treatment include persons with mild cognitive impairment or mild dementia due to AD (Clinical Stages 3–4, MMSE 20–30) who have biomarker confirmation of AD pathology by PET or CSF. Tau PET is not required for eligibility. Apolipoprotein E (*APOE*) genotyping should be performed prior to treatment to inform an individual’s risk of developing Amyloid-Related Imaging Abnormalities (ARIA). Pre-treatment MRI should be obtained no more than 12 months prior to treatment. Patients with findings of >4 cerebral microbleeds, cortical superficial siderosis or a major vascular contribution to cognitive impairment should be excluded from treatment. The decision to initiate therapy should be grounded in a shared decision-making process that emphasizes the patient’s values and goals of care. Donanemab is administered as a monthly intravenous infusion. Surveillance MRIs to evaluate for ARIA should be performed prior to the 2nd, 3rd, 4th and 7th infusions, prior to the 12th dose in higher risk individuals, and at any time ARIA is suspected clinically. Clinicians may consider discontinuing treatment if amyloid clearance is demonstrated by amyloid PET, typically obtained 12–18 months after initiating treatment.

## 1. Introduction

Donanemab (Kisunla^®^) is an IgG1 monoclonal antibody directed at the N-terminally truncated, pyroglutamate-modified forms of amyloid-*β* (A*β*) present on mature amyloid plaques [1]. Treatment with donanemab leads to macrophage-mediated clearance of A*β* plaques in transgenic Alzheimer’s disease (AD) mouse models [1] and in human clinical trials [2,3]. In the Phase 2 double-blinded, randomized placebo-controlled trial (RCT) TRAILBLAZER-ALZ (ClinicalTrials.gov identifier: NCT03367403), 257 patients with MCI/mild dementia due to AD, positive amyloid PET and low-medium tau burden on tau PET were randomized to receive monthly intravenous infusions of donanemab or placebo for 76 weeks [2]. Treatment with donanemab was associated with 32 % slowing of decline on the primary clinical endpoint, the Integrated Alzheimer’s Disease Rating Scale (iADRS). Donanemab treatment reduced A*β*-PET uptake by a mean of 85 Centiloids (CL). The confirmatory TRAILBLAZER-ALZ2 Phase 3 RCT (NCT04437511) enrolled 1736 participants with MCI/mild dementia due to AD who were amyloid PET-positive (68 % with low-medium tau PET uptake, 32 % with high tau PET) [3]. Compared to placebo, treatment with donanemab at 76 weeks was associated with 35 % slowing of decline on the iADRS (primary endpoint) in the low-medium tau group, and 22 % slowing on the iADRS in the combined population (including patients with high tau PET). Consistent results, with significant slowing were similarly observed on all pre-specified secondary clinical endpoints, including the Clinical Dementia Rating – Sum of Boxes (CDR-SB; 36 % slowing in the low-medium tau group, 29 % slowing in the combined population). Amyloid PET was reduced by a mean of 88 CL compared to placebo in the combined population. Based on these data, the United States (U.S.) Food and Drug Administration (FDA) approved donanemab in July 2024 for the treatment of patients with MCI or mild dementia due to AD and biomarker-confirmed A*β* pathology [4].

Donanemab recognizes an N-terminally truncated form of A*β* that lacks aspartate-1 and alanine-2 and has a cyclized pyroglutamate (pE) as its third amino acid (termed “N3pE-A*β*”) [5]. This modified form of A*β* constitutes a small minority (~1–3 %) of A*β* peptides in AD brain tissue [1]. N3pE-A*β* is insoluble and believed to occur exclusively in fibrous amyloid plaques and cerebrovascular deposits, not in aqueously diffusible A*β* oligomers and protofibrils. Analogous to antibodies to the native N-terminus (e.g., lecanemab), donanemab crosses the blood brain barrier after intravenous administration and binds to N3pE-A*β* in plaques and blood vessels. Antibody binding to either form of A*β* is believed to stimulate local microglial cells to phagocytose the A*β*-antibody complexes and thereby degrade and clear amyloid deposits over time. Since the N3pE variant comprises < 3 % of A*β* peptides in most AD brains, it is assumed that the far more abundant A*β* peptides that begin with aspartate-1 are also phagocytosed by the activated microglia, a process referred to as “bystander clearance.” Given the low abundance of N3pE-A*β* in AD brain, the donanemab clinical trial design included an opportunity to stop treatment once the antigen had presumably been cleared, i.e., if plaques become undetectable by amyloid PET.

A*β*-targeting monoclonal antibodies have resulted in a completely new paradigm of AD care which represents new opportunities but also challenges due to gaps in knowledge, interdisciplinary coordinated care pathways and healthcare system resources. Similar to previously published Appropriate Use Recommendations (AUR) for the A*β*-targeting antibodies aducanumab [6] and lecanemab [7], the donanemab AUR presented in this manuscript provide consensus-based, expert recommendations on the translation of the drug into real-world patient care. Real-world patients with early-stage AD tend to be older, more diverse, less well educated, and have greater rates of comorbidities and higher concomitant medication use than clinical trial participants [8]. Real-world patients are also treated by clinicians who are less familiar and experienced with the use of novel drugs such as A*β*-targeting monoclonal antibodies, and in healthcare systems that are less well-resourced and coordinated, compared to clinical trial programs, for optimal management and monitoring of these drugs [8]. Furthermore, TRAILBLAZER-ALZ and TRAILBLAZER-ALZ2 included innovative aspects in the clinical trial design (e.g., tau PET for eligibility and stratification, amyloid PET based stopping criteria) that may be difficult to replicate in real-world clinical care. The goal of the donanemab AUR is to assist clinicians in the appropriate and safe use of this novel therapy. The AUR are intended to complement clinical trial publications, the FDA prescribing information and sponsor-created marketing information, by providing an independent, academic perspective on appropriate use of donanemab, with an emphasis on maximizing patient safety. The AUR do not provide endorsement for treatment, evaluate meaningfulness related to efficacy or effectiveness, or consider costs and reimbursement. They should be viewed as recommendations, not guidelines, criteria or requirements. The AUR are intended to be a “living document” that evolve as additional data emerge to inform real-world use of donanemab. Above and beyond the AUR, clinical judgment should always be considered paramount when evaluating the suitability of an individual patient to receive or continue this novel therapy.

## 2. Method

The donanemab AUR were developed by the AD and Related Disorders Therapeutic Work Group (ADRD TWG) and additional invited *ad hoc* experts. The methodology was similar to that used to develop AUR for aducanumab and lecanemab [6,7]. Work Group members reviewed all publicly available data regarding the drug’s efficacy and safety profile, including the published clinical trial results from TRAILBLAZER-ALZ and TRAILBLAZER-ALZ2, the open-label safety addendum to TRAILBLAZER-ALZ2, FDA and sponsor materials presented to the FDA Peripheral and Central Nervous System Drugs Advisory Committee (convened on June 10, 2024) and the FDA prescribing information. In formulating its recommendations, the Work Group considered the totality of available safety data pertaining to other A*β*-targeting antibodies, as well as expert opinion. Work Group deliberations took place between October 2023 and November 2024 via video conferences and written exchanges, with reiterative refinement of AUR until group consensus was achieved. All listed authors individually approved this final published version of the AUR.

## 3. Appropriate patient for treatment with donanemab

### 3.1. Clinical characteristics

The core clinical eligibility criteria for the AUR are grounded in the inclusion and exclusion criteria for TRAILBLAZER-ALZ2, since this is the population in which the safety and efficacy of donanemab have been demonstrated [3]. Donanemab is indicated for treatment of patients in the early symptomatic stages of AD, i.e., those at the MCI or mild dementia stages of disease. The clinical criteria for MCI and mild dementia are presented in Table 1. In the recently revised Alzheimer’s Association Workgroup criteria for diagnosis and staging of AD [9], MCI is equivalent to Clinical Stage 3 (cognitive impairment with early functional impact), and mild dementia is synonymous with Clinical Stage 4 (dementia with mild functional impairment). Donanemab treatment should not be initiated in patients with moderate-to-severe dementia (i.e., those requiring assistance in basic activities of daily living, Clinical Stage ≥ 5), as the safety and efficacy of the drug have not been established in this more advanced disease stage. Similarly, donanemab treatment is not currently indicated in patients in earlier clinical stages of AD, including those with biomarker evidence of A*β* pathology but no symptoms (Clinical Stage 1), or those who have subjective cognitive complaints but perform normally in daily activities and on formal neuropsychological testing (Clinical Stage 2). These recommendations may be updated in the future depending on the results of ongoing clinical trials of donanemab in these earlier-stage populations (e.g., TRAILBLAZER-ALZ3, NCT05026866).

To be considered as candidates for treatment, AD should be suspected as the primary etiology of cognitive impairment following a comprehensive clinical evaluation, as recommended in recently published clinical practice guidelines [11,12]. While most patients included in TRAILBLAZER-ALZ2 presented with a typical amnestic phenotype, the AUR allow for inclusion of patients presenting with non-amnestic cognitive-behavioral syndromes strongly linked to AD neuropathology [11], including posterior cortical atrophy (PCA, presenting with primary visuospatial dysfunction) [13], the logopenic-variant of primary progressive aphasia (lvPPA, presenting with primary language deficits) [14] and dysexecutive AD [15], based on strong evidence for shared pathophysiology across these AD phenotypes [16]. Conversely, patients presenting with cognitive-behavioral syndromes suggestive of non-AD etiologies (e.g., dementia with Lewy bodies, behavioral-variant frontotemporal dementia, nonfluent and semantic variants of PPA) would rarely be candidates for donanemab, as positive A*β* biomarkers would more likely be indicative of AD co-pathology than AD as the primary cause of impairment [11,17]. Patients in whom alternative medical, psychiatric or neurological etiologies are suspected to be the primary cause of cognitive impairment should likewise be excluded from treatment. Ultimately, clinical judgment is paramount in determining whether a patient’s symptoms are primarily driven by AD pathology and thereby render the patient a candidate for donanemab treatment.

Although they were not explicitly excluded from TRAILBLAZER-ALZ or TRAILBLAZER-ALZ2, patients with autosomal dominant AD (ADAD) (i.e., patients with disease-causing mutations in amyloid precursor protein (*APP*), presenilin-1 (*PSEN1*) and presenilin-2 (*PSEN2*) genes) likely represent a tiny minority of all treated patients in the clinical trials. Therefore, the efficacy and safety of donanemab in this population is unknown. In evaluating a patient with ADAD for donanemab treatment, clinicians should consider the patient’s specific mutation, and specifically the degree to which the mutation is associated with prevalence and burden of cerebral amyloid angiopathy (CAA) [18,19] (see Alzforum.org for a continuously updated ADAD mutation database). The Work Group recommends *excluding* patients with *APP* mutations associated with severe CAA [20], given concerns for potentially higher risks and complications of amyloid related imaging abnormalities (ARIA). Patients with other ADAD mutations that are not known to be associated with severe CAA may be considered for donanemab treatment, assuming they meet other inclusion/exclusion criteria, and acknowledging uncertainties about risks and benefits of treatment in this population. Similarly, the Work Group recommends excluding patients with Down syndrome related AD, who also show a higher prevalence and severity of CAA compared to patients with sporadic AD [19,21]. Referral of eligible patients with ADAD and AD due to Down syndrome to clinical trials specifically recruiting these populations (e.g., the Dominantly Inherited Alzheimer Network Trials Unit (DIAN-TU) and the Trial-Ready Cohort-Down Syndrome (TRC-DS) program) should be strongly considered in order to better establish the safety and efficacy of A*β*-targeting treatments and other therapeutic targets in these genetically-driven sub-types of AD.

Table 2 lists additional core eligibility criteria in TRAILBLAZER-ALZ2 side-by-side with the AUR. Patients included in TRAILBLAZER-ALZ2 were between 60 and 85 years-old at enrollment. Physician judgment should be used for treating patients outside this age range. In most cases, baseline Mini Mental State Exam (MMSE) scores of appropriate patients will fall in the range of 20–30, similar to the MMSE range of 20–28 used for trial inclusion. We extended the upper limit of the MMSE to 30 since, in clinical practice, patients can fully meet criteria for MCI (including showing objective evidence of impairment on cognitive testing), but still achieve a perfect score on a global screening tool such as the MMSE. The lower limit MMSE score of 20 is roughly equivalent to a score of 11–13 out of 30 points on the Montreal Cognitive Assessment (MoCA) [22,23]. Clinical judgment is warranted where specific patient-related factors may confound performance on the MMSE, MoCA, or other brief, standardized and validated tools used to measure global cognition [24]. For example, low educational attainment, lack of proficiency in the language of the test, or atypical clinical presentations of AD (e.g., lvPPA) may lead to lower MMSE scores that may not be indicative of a more advanced disease stage.

The Work Group recommends that patients and clinicians identify a care partner, family member or friend who can provide the patient with the support needed for donanemab treatment. The specific roles of the care partner will vary from patient to patient depending on individual circumstances, but may include assistance with decision making about diagnostic testing and treatment options, logistical support in coordinating and implementing care (e.g., managing the appointment schedule, assisting with transportation to and from appointments, following through on treatment recommendations), monitoring for and alerting the treating clinician about adverse events, and supporting the patient in all other aspects of care and daily activities as appropriate.

### 3.2. Biomarker confirmation of A*β* pathology

Biomarker confirmation of A*β* pathology by PET or CSF is required for patients to be eligible for treatment with donanemab. In TRAILBLAZER-ALZ2, eligibility was determined using amyloid PET interpreted via a central quantitative read [3], set at a threshold of ≥ 37 CL [26]. However, in clinical practice, amyloid-PET is interpreted using FDA-approved visual read methods that provide a binary read of “positive/elevated” or “negative/non-elevated” A*β* plaques based on cortical tracer retention [27]. PET scans should be performed using an FDA-approved A*β* radiotracer (^18^F-florbetaben (Neuraceq^®^), ^18^F-florbetapir (Amyvid^®^), ^18^F-flutametamol (Vizamyl^®^)). Though there are FDA-approved software packages that enable Centiloid quantification of A*β*-PET, these are not yet routinely used in clinical practice. The AUR consider visual reads of amyloid PET with an FDA-approved tracer to be sufficient to establish eligibility for treatment. Notably, the quantitative threshold of 37 CL used in the clinical trials was intended to enrich the trials for participants with more advanced disease, who were also likely to show elevated binding on tau-PET (see below). In clinical practice, positive visual reads of amyloid PET correspond to a slightly lower quantitative threshold (~25 CL) [28–30], such that eligibility based solely on visual read will result in treatment of some patients with lower amyloid plaque burden than those included in the donanemab clinical trials.

Alternatively, treatment eligibility can be determined by CSF AD biomarkers. To date, three CSF tests have been approved by the FDA for determination of amyloid status: A*β*42/A*β*40 ratio measured on the Lumipulse platform, and p-tau181/A*β*42 or total tau/A*β*42 ratios measured on the Elecsys platform. These CSF biomarker ratios have approximately 90 % sensitivity and specificity when compared to amyloid PET [31–33] or clinically significant AD pathology post-mortem [34,35]. Importantly, CSF A*β*42 by itself is inferior to the CSF biomarker ratios in classification of amyloid PET status [31]. Therefore, the validated and FDA-approved biomarker ratios should be used as the primary assay readout to determine amyloid status in patients being considered for donanemab treatment. CSF AD biomarker ratios and amyloid PET are considered equivalent in terms of accuracy and performance, and the choice of amyloid biomarker may be driven by clinic and PET imaging access, patient preference and reimbursement considerations. However, only amyloid PET is validated to assess amyloid plaque clearance in response to treatment (see “Amyloid PET Based Discontinuation of Treatment” section below).

Blood biomarker tests for AD pathology are rapidly developing [36,37]. These tests use mass spectrometry or high sensitivity immunoassays to measure concentrations of A*β* peptide fragments and phosphorylated tau (typically at positions 217 or 181) in plasma. Similar to findings in CSF, reductions in the plasma A*β*42/A*β*40 ratio or increases in the plasma concentration of p-tau217 and p-tau181 are associated with the presence of amyloid pathology, with the highest performing tests showing similar accuracy to CSF biomarker tests when compared to amyloid PET [38]. Blood biomarkers have great potential to enhance access, convenience and affordability of AD biomarker testing. However, there are still barriers to implementation of blood-based biomarkers in routine clinical practice [37,39,40]. First, there is currently significant variability in the accuracy and degree of validation of clinically available AD blood biomarker assays [41]. Clinical experience with these tests is limited, and their performance in real-world populations [42,43], and in particular in racially and ethnically diverse cohorts [44,45], is not yet well established. Importantly, none of the clinically available tests are to date approved by the FDA for clinical use. Weighing all these factors, the Work Group does not recommend establishing eligibility for treatment based solely on a positive AD blood biomarker test at this time. However, we anticipate that specific AD blood biomarker tests will soon meet the rigorous FDA criteria for clinical approval, and that the high-performing tests that achieve regulatory approval and meet performance criteria specified in a recent consensus statement [37] will be appropriate to use for establishing eligibility for treatment with donanemab and other A*β*-targeting therapies.

### 3.3. Tau PET

Tau PET played a key role in the inclusion criteria for TRAILBLAZER-ALZ2 [3]. Participants were excluded from the trial if they showed no evidence of neocortical tau tracer uptake based on visual assessment. Patients who showed neocortical tau PET retention were stratified, based on a combination of visual assessment and image quantification, into those with low/medium tau (68 % of all randomized participants), versus those with high tau (32 % of all randomized participants). Drug efficacy was measured separately in the “low/medium tau” group and in a “combined population” that included both “low/medium” and “high tau” participants. Overall, there was greater slowing of clinical decline in the “low/medium tau” group than in the “combined population” across primary and secondary clinical endpoints, suggesting greater clinical benefit from donanemab for those treated at earlier stages of tau deposition [3].

At present, tau PET is primarily a research tool. Clinical access to the one FDA-approved tau PET radiotracer, ^18^F-flortaucipir (Tauvid^®^) [46], is extremely limited in the United States. Furthermore, the FDA-approved method for tau PET interpretation is based on a binary visual read [46], and clinically available methods for tau PET quantification (as applied in TRAILBLAZER-ALZ2 for patient stratification) are lacking. For these reasons, the Work Group concluded that tau PET should *not* be required for eligibility for donanemab treatment. If available, tau PET can be used to individualize the estimate of clinical benefit for a particular patient, thus informing risk/benefit discussions.

Given the limitations noted above, it is expected that the majority of patients receiving donanemab in the near future will have unknown tau PET status. This will result in treatment of some patients who would have been excluded from the clinical trial due to “no/very low” tau PET uptake. It is important to recognize that the clinical benefit of donanemab has not been definitively established in such patients, though current indications from trials of other A*β*-targeting antibodies suggest a significant clinical benefit in no/very low tau PET participants [47]. Safety data have been collected in patients who were not subjected to the tau PET requirement as part of the open-label addendum to TRAILBLAZER-ALZ2 (see below section on ARIA) [48].

### 3.4. Concomitant medications

In evaluating the use of concomitant medications with donanemab treatment, the Work Group considered whether medications may compromise patient safety, as well as whether medications were exclusionary from participation in the phase 3 clinical trial. Patients taking standard anti-dementia therapies that act on neurotransmitter systems (i.e., cholinesterase inhibitors and memantine) were allowed in the phase 3 clinical trial, and it is appropriate to prescribe donanemab on the background of these symptomatic therapies. Patients requiring treatment for psychiatric illnesses may be considered for treatment with donanemab if the psychiatric condition and the related medications do not compromise the ability of the patient to understand or be adherent to the schedule of required infusions, scans, and clinical assessments.

Lecanemab (Leqembi^®^) and aducanumab (Adulhelm^®^) are anti-amyloid monoclonal antibodies approved for the treatment of early AD. These agents are in the same therapeutic class as donanemab and their use is approved for their initiation in the same early AD population. The administration and management of aducanumab and lecanemab differ from that of donanemab [6,7], and patients concurrently receiving aducanumab or lecanemab should not be treated with donanemab. Patients who were treated with one of these agents in the past and are now off therapy might be considered for donanemab treatment (see “Switching to Donanemab from Another A*β*-Targeting Monoclonal Antibody” section below); caution should be exercised if the individual had complications with use of these agents, such as the occurrence of ARIA.

The use of anti-thrombotics and their association with ARIA risk is discussed in detail in the “ARIA” section below. In brief, the AUR allow the concomitant use of aspirin (up to 325 mg/day) or other antiplatelet agents (e.g., clopidogrel, prasugrel, ticagrelor) at standard therapeutic doses when used as monotherapy. The Work Group was not able to offer a recommendation regarding patients on dual anti-platelet therapy due to a paucity of safety data. The AUR recommend *against* the use of donanemab in patients receiving anticoagulants, including warfarin, heparin, and direct oral anticoagulants (e.g., dabigatran, rivaroxaban, edoxaban, apixaban, betrixaban) until additional safety data are available. Anticoagulants should *not* be discontinued for the purposes of initiating donanemab if anticoagulation is otherwise medically indicated. As always, clinical judgment is paramount in weighing the relative risks and benefits of offering donanemab treatment versus continued administration of anticoagulants and other exclusionary concomitant medications.

The Work Group recommends that patients receiving donanemab *not* receive treatment with tissue plasminogen activator (tPA), tenecteplase (TNK), or other thrombolytic agents, given reports of fatal ARIA-E and ARIA-H associated with intravenous thrombolysis in patients receiving donanemab and other A*β*-targeting monoclonal antibodies [48,49]. Mechanical thrombectomy without thrombolytics may be an alternative in appropriate scenarios [50] (see “ARIA” section below).

There is growing evidence that ARIA represents a treatment-induced variant of cerebral amyloid angiopathy-related inflammation (CAA-ri), an inflammatory vasculopathy that is thought to have an autoimmune etiology [49–53]. It is unclear if the presence of other autoimmune disorders increases an individual’s risk of developing ARIA, and the impact of chronic immunosuppressive therapy on the efficacy of donanemab is also unknown. Until more data are available, the Work Group recommends excluding patients with immunologic disorders requiring therapy with immunoglobulins, monoclonal antibodies, immunosuppressants, or plasmapheresis. A history of seizures may increase the risk of seizures and status epilepticus in association with ARIA, and we recommend excluding patients with a history of seizures until additional safety information is available. Poorly controlled hypertension represents a risk factor for ARIA, with patients with mean arterial pressure > 93 mm Hg at elevated risk compared to those with lower mean arterial pressure [48]. Therefore patients should not be treated with donanemab unless their blood pressure is controlled. Patients with a history of stroke or TIA within 12 months should be excluded. Similarly, we recommend that patients with bleeding disorders and other unstable medical or psychiatric conditions be excluded from treatment with donanemab; they may become therapy candidates if medical and psychiatric comorbidities are controlled.

### 3.5. MRI findings

Eligible patients must have a screening MRI within at most 12 months (and ideally <6 months) of treatment initiation. The goal of the baseline MRI is to screen for imaging evidence of CAA, which increases the risk of ARIA, and to identify patients in whom a high burden of cerebrovascular disease or other structural imaging findings suggest that non-AD etiologies are significantly contributing to cognitive symptoms. Patients who are unable to undergo MRI due to claustrophobia, pacemaker, defibrillator, or metal implants are not eligible for donanemab therapy. Computerized tomography (CT) of the brain is *not* adequate to screen for exclusionary imaging findings or to monitor for ARIA once treatment is initiated.

A non-contrast MRI, utilizing TI fluid-attenuated inversion recovery (FLAIR) and T2* -weighted Gradient Recalled Echo (GRE) or other heme-sensitive sequences (such as susceptibility weighted imaging (SWI)), and diffusion weighted imaging (DWI), preferably on a 3T magnet should be obtained to determine if an individual is a candidate for donanemab therapy [54]. 1.5T MRI is acceptable if 3T MRI is not available, though is less sensitive in detecting white matter hyperintensities and hemosiderin deposits. The primary MRI-based exclusion criteria are listed in Table 2. The AUR MRI exclusion criteria largely align with those implemented in TRAILBLAZER-ALZ2. Patients with one region of cortical superficial siderosis (cSS) at baseline were included in TRAILBLAZER-ALZ2 (as well as clinical trials of the A*β*-targeting antibodies gantenerumab and trontinemab [55,56]) but were excluded from the aducanumab and lecanemab clinical trials. It has subsequently been shown that a single region of cSS increases the risk of ARIA-E [48]. There have also been reports of severe and fatal ARIA among patients with one region of cSS on the baseline MRI [3,55]. Based on these findings, the Work Group recommends excluding patients with a single region of cSS on their baseline MRI from treatment. The Work Group additionally recommends excluding the rare patients who meet MRI criteria for spontaneous CAA-ri on baseline MRI (Table 3). If patients show borderline findings (e.g., 1–4 cortical microbleeds) or are at higher risk for ARIA (e.g., homozygous for apolipoprotein E *ε*4 (*APOE4*)) and the baseline MRI was obtained > 6 months prior to initiating treatment, a repeat MRI may be strongly considered to ensure that imaging findings have not progressed and crossed the AUR threshold for exclusion.

In clinical practice, we recommend operationalizing the exclusionary criterion of “severe white matter disease” as a score of 3 on the Fazekas Scale (or a similar score on a standardized and validated white matter hyperintensity scale, such as a score of 3 on the European Task Force Age-Related White Matter Changes scale [57]), which is characterized by large confluent hyperintensities in the deep white matter or irregular periventricular signal extending into deep white matter [58]. In addition to the MRI exclusion criteria, we recommend excluding patients with MRI evidence of a significant vascular contribution to cognitive impairment and dementia. This includes patients with evidence of territorial infarcts > 1 cm, and patients with > 2 lacunar infarcts. Clinical judgment is central in determining the relevance of vascular lesions on MRI to the patient’s cognitive deficits. Additionally, the risk of ARIA in patients with cerebral contusion, encephalomalacia, brain aneurysms or other vascular malformations, central nervous system infection and brain tumors is unclear as these patients were excluded from clinical trials. The Work Group recommends against treating these patients until more safety data are available. Conversely, the presence of small meningiomas, arachnoid cysts and small venous anomalies did not exclude patients from the donanemab trials, and the Work Group agreed these findings should not be considered exclusionary.

### 3.6. APOE genotyping

As with other A*β*-targeting antibodies, there is a strong, dose-dependent association between the number of apolipoprotein E *ε*4 (*APOE4*) alleles and the risk of ARIA in patients treated with donanemab (see “ARIA” section for details) [3,48]. Recurrent, symptomatic and severe ARIA were also more common in *APOE4* carriers. Based on these data, the FDA issued a boxed warning on using donanemab in *APOE4* homozygotes [4]. Given the strong association between *APOE* genotype and ARIA risk, the Work Group recommends that *APOE* genotyping be performed prior to initiating donanemab treatment, and that specific ARIA risks based on *APOE* genotype be discussed with patients and care partners as part of the shared decision-making process when considering treatment [59]. No *APOE* tests are currently FDA approved, but many clinical laboratories offer genotyping. Some laboratories determine *APOE* genotype indirectly by assessing the patient’s proteotype, which is also acceptable. Providers should confirm that tests are performed in accordance with appropriate standards, i.e., in laboratories approved by Clinical Laboratory Improvement Amendments (CLIA). Providers should counsel patients about the implications of *APOE* genotyping results, including implications for risk of AD for their family members, before ordering testing [60].

## 4. Appropriate dosing, administration and monitoring of donanemab

### 4.1. Administration of donanemab

**Fig. 1.**
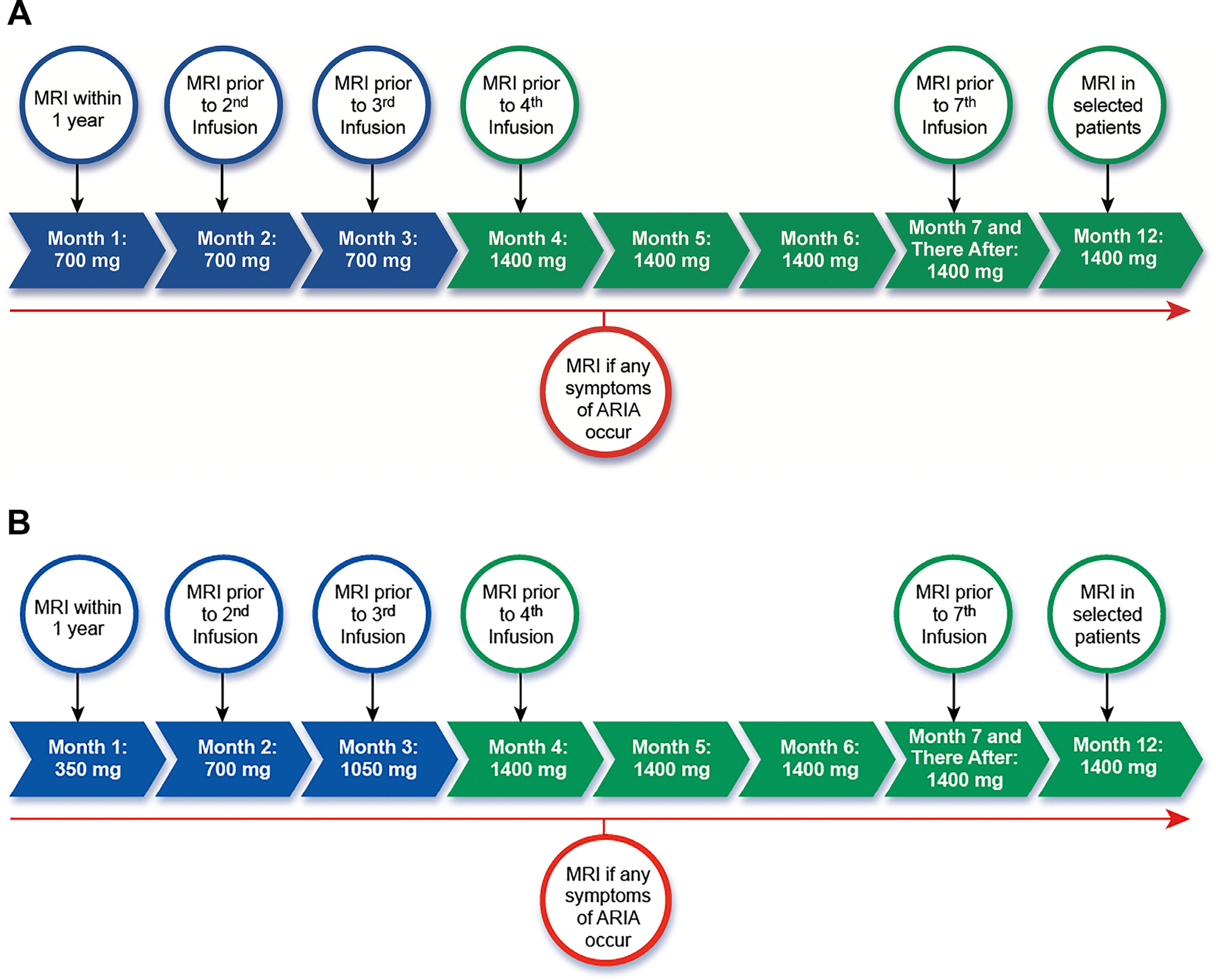
Dose titration and MRI monitoring schedule for donanemab. A) Dose titration used in TRAILBLAZER-ALZ2 and approved by the FDA. B) Adjusted titration schedule from TRAILBLAZER-ALZ6 found to be associated with a reduced rate of ARIA.

Donanemab is administered intravenously every four weeks. The drug is provided in 350 mg/20 mL vials and is diluted prior to administration with 0.9 % saline to a target of 4–10mg/mL (dilute with 70–175 mL of saline solution for 700 mg dose; 140–350 mL of saline solution for 1400 mg dose). The recommended dosing is 700 mg every four weeks for three doses, followed by 1400 mg every 4 weeks there-after (Fig. 1A), following the dose titration performed in the phase 3 trial and prescribing information. The entire diluted solution should be administered over 30 min. We recommend that patients be observed for 1 hour after the first four infusions to observe for infusion and hypersensitivity reactions (see “Infusion Reaction” section). The observation period may be reduced to 30 min beginning with the fifth infusion, if no infusion reactions have occurred or if it has been established that infusion reactions can be prevented with prophylaxis.

In a follow-up, open-label clinical trial (TRAILBLAZER-ALZ6, NCT# NCT05738486) in patients with early-stage symptomatic AD, patients were randomized to receive standard dosing or one of three modified dose titration schedules in order to evaluate if risk of ARIA can be mitigated by alternative dosing regimens [61]. After 6 months of follow-up, the lowest rates of ARIA were observed in patients treated with a modified titration schedule (350 mg at first dose, 700 mg at second dose, 1050 mg at third dose, 1400 mg every 4 weeks thereafter). Overall rates of ARIA-E were 14 % in the modified titration arm, compared with 24 % in the standard titration arm. Rates of ARIA-E in *APOE4* homozygotes were lower in the modified titration arm (19 %) than in the standard titration arm (57 %), as were overall rates of symptomatic ARIA-E and the radiographic severity of ARIA, while amyloid lowering on PET was similar in both arms. Important caveats are the relatively small sample sizes (*N* = 212 in modified titration arm and *N* = 207 in standard titration arm) and the limited duration of follow-up data available thus far (6 months). With these caveats in mind, and noting that the modified dosing regimen has not yet been reviewed or approved by the FDA, prescribing clinicians may choose to incorporate the modified titration regimen into their practice with the intent of mitigating the risk of ARIA, particularly in high-risk individuals (Fig. 1B).

## 5. Amyloid related imaging abnormalities (ARIA): Frequency, risk factors and monitoring

ARIA, a common adverse effect of treatment with donanemab and other A*β*-targeting monoclonal antibodies, can take two forms. ARIA with edema (ARIA-E) leads to edematous changes in gray and white matter, whereas ARIA with hemorrhage (ARIA-H) presents with microhemorrhages, convexity subarachnoid hemorrhage, cSS, and (very rarely) macrohemorrhages. The mechanisms underlying ARIA are still being elucidated, but likely involve an interaction between A*β*-targeting antibodies and vascular amyloid deposits in CAA-affected vessels, leading to inflammation and hemorrhage [48,50,53]. Spontaneous CAA-related microbleeds are relatively common in patients with AD, but spontaneous CAA-related inflammation (CAA-ri) is rare in the absence of amyloid lowering treatment. ARIA often manifests purely as a neuroimaging abnormality, though approximately 25 % of ARIA events are accompanied by symptoms, which are often mild-moderate but can rarely be severe (see below). ARIA is detected by MRI performed either as part of routine screening during treatment (Fig. 1A, B) or when symptomatic ARIA is suspected. ARIA-E is best detected on FLAIR sequences, whereas GRE or SWI sequences detect ARIA-H [54].

The frequency of ARIA-E and ARIA-H by *APOE4* genotype in the donanemab placebo-controlled trials (TRAILBLAZER-ALZ and TRAILBLAZER-ALZ2) and open-label addendum are shown in Table 4. In the placebo-controlled trials, the overall frequency of ARIA in donanemab-treated patients was 37.0 % compared to 14.2 % in patients receiving placebo [2,3,48]. ARIA-E rates were 24.4 % with donanemab treatment vs. 1.9 % with placebo, whereas ARIA-H rates were 31.3 % with treatment vs. 13.0 % with placebo. Rates of isolated ARIA-H were similar with treatment (12.5 %) and placebo (11.7 %), with excess ARIA-H cases in the treatment group attributed to co-occurring ARIA-E and ARIA-H. Rates of ARIA-E (19.8 %) and ARIA-H (27.2 %) were slightly lower in the open-label addendum, possibly due to slightly lower baseline amyloid burden (~20 CL lower mean baseline amyloid PET in participants in the open-label addendum versus those in the placebo-controlled trials). The majority of overall ARIA-E and ARIA-H events (and nearly all serious ARIA events) occurred very early in treatment (typically within the first 3 doses), prompting the recommended monitoring schedule of safety MRIs prior to the 2nd, 3rd, 4th and 7th infusions (Figure 1A, B). This schedule is consistent with the amended TRAILBLAZER-ALZ2 protocol (MRI was added prior to the second infusion) and the FDA prescribing information. Unscheduled urgent MRIs should be obtained to evaluate new symptoms concerning for ARIA, as discussed below. We also recommend considering an MRI scan after one year of treatment, prior to the 12th dose, in higher-risk individuals (e.g., *APOE4* carriers, patients with previous ARIA events earlier in treatment). Whenever possible, the pre-treatment MRI and all safety MRIs should be performed on the same scanner (and at minimum using the same field strength) to facilitate comparisons between images acquired at different timepoints [54].

Symptoms associated with ARIA range from non-specific and mild (e.g., headache, increased confusion, dizziness, nausea, imbalance, gait or vision changes) to serious neurological sequelae such as seizures, encephalopathy and focal neurological deficits mimicking an acute stroke (Table 5) [3,51,62]. Collectively, 99/447 (22.1 %) of ARIA events were symptomatic in the donanemab placebo-controlled trials and open-label addendum [48]. The most common symptoms associated with ARIA were headache and confusional state. Out of 52 symptomatic ARIA events in TRAILBAZER-ALZ2, symptoms were rated as mild in 30, moderate in 12 and severe in 10 participants. Overall, symptom resolution occurred within the study period in 80 % of symptomatic cases in the placebo-controlled trials. In addition to the routine surveillance MRI schedule, any concern for ARIA-related symptoms should prompt an urgent or emergency MRI scan (depending on the nature or severity of symptoms), and patients should not receive their next dose of donanemab until ARIA has been definitively ruled-out.

As has been observed with other potent amyloid-lowering antibodies [62,63], the primary risk factor for ARIA in the setting of donanemab therapy is *APOE4* genotype. The rates of ARIA-E in the placebo-controlled trials were 14.8 % in *APOE4* non-carriers, 24.1 % in *APOE4* heterozygotes and 41.7 % in *APOE4* homozygotes, while ARIA-H rates were 18.9 % in *APOE4* non-carriers, 31.0 % in *APOE4* heterozygotes and 53.6 % in *APOE4* homozygotes (Table 4). Rates of symptomatic ARIA-E were 4.1 %, 6.1 % and 7.7 % amongst *APOE4* noncarriers, heterozygotes and homozygotes respectively in the placebo-controlled trials, with serious and severe ARIA also being most common amongst *APOE4* homozygotes. Given the elevated risk in this population, the FDA placed a boxed warning on use of donanemab in *APOE4* homozygotes [4]. The AUR do not exclude *APOE4* homozygotes from donanemab treatment, but strongly recommend that clinicians proceed with caution in this population. A thorough and careful discussion of risks and benefits of treatment, including direct discussion of the higher risks of ARIA and a comprehensive evaluation for additional ARIA risk factors (see below), is especially critical as part of the shared decision-making process in this population. There have been at least 2 cases of intracerebral hemorrhages in patients with an *APOE2* allele being treated with lecanemab [64] and trontinemab [55]. This is noteworthy since, although the *APOE2* allele reduces the risk of developing AD, it has been associated with *increased* risk of CAA-related hemorrhage and cSS [65,66]. While the association between *APOE2* and ARIA risk in the setting of A*β*-targeting therapy remains unclear, there is at least a hypothetical concern for increased risk of hemorrhage, warranting additional caution in screening and counseling of these individuals.

In addition to *APOE4* genotype, a post-hoc analysis of donanemab-treated patients identified the number of baseline microhemorrhages, presence of baseline superficial siderosis, elevated mean arterial pressure (≥ 93 mm Hg) and higher baseline amyloid PET burden as independent predictors of ARIA-E, whereas antihypertensive use was found to be protective [48]. Of these, elevated blood pressure deserves special consideration as a potentially modifiable risk factor for ARIA, highlighting the importance of ensuring adequate blood pressure control in hypertensive patients prior to initiating donanemab treatment. The presence of a single cortical microbleed was associated with a higher risk of ARIA-E compared to patients with no microbleeds, and this risk was further heightened in patients with 2–4 microbleeds. Therefore, even within the narrow range of 0–4, the number of baseline microbleeds should be considered when evaluating an individual patient’s ARIA risk. Similarly, baseline amyloid PET values ≥ 108 CL were associated with a slightly increased risk of ARIA-E.

### 5.1. Cerebral macrohemorrhages

Cerebral macrohemorrhages represent a rare but very serious manifestation of ARIA-H, or a spontaneous event associated with CAA (as well as other vascular risk factors). Intracerebral macrohemorrhages have high mortality and morbidity, with patients at risk of having enduring neurological disability [67]. Overall, macrohemorrhages occurred in 3/984 (0.3 %) donanemab-treated and 2/999 (0.2 %) placebo-treated individuals in the placebo-controlled trials and 4/1047 (0.4 %) donanemab-treated individuals in the open-label addendum [48]. One participant in TRAILBLAZER-ALZ2 developed a fatal intracerebral hemorrhage after 2 doses of donanemab [3]. Notably, this participant had a large area of baseline cSS on pre-treatment MRI. One patient in the long-term addendum and one patient in the modified titration study developed a fatal intracerebral hemorrhage after receiving thrombolytic treatment for a suspected acute stroke [3,61]. Further clinical description of macrohemorrhages in donanemab-treated patients is provided in Zimmer et al. [48]. A similar number of ARIA-related fatalities has been reported with lecanemab in open-label studies and early clinical use [68,69].

### 5.2. Risk of ARIA and anti-thrombotic use

Rates of ARIA-E, ARIA-H and intracerebral hemorrhages in the donanemab placebo-controlled trials, stratified by use of antithrombotics and anticoagulants, are shown in Table 6. There was no elevated risk of ARIA in patients treated with anti-platelets, and therefore the Workgroup recommends allowing patients on standard doses of monotherapy with aspirin (up to 325 mg/day) or other antiplatelet agents (e.g., clopidogrel, prasugrel, ticagrelor) at standard therapeutic doses to be considered as treatment candidates for donanemab if they meet other criteria for therapy. There were insufficient safety data available for the Work Group to make a recommendation about donanemab use in patients on dual anti-platelet therapy.

There are limited data to inform the safety of donanemab use in patients requiring chronic anticoagulation. Anticoagulants, including warfarin, vitamin K antagonists, direct oral anticoagulants (e.g., dabigatran, rivaroxaban, edoxaban, apixaban, betrixaban), or heparin may confer an increased risk for macrohemorrhage associated with ARIA [7,70]. While there did not appear to be an increased risk of ARIA-H or macrohemorrhage in patients on anticoagulants compared to those who were not anticoagulated in the donanemab trials, the total number of patients on anticoagulation was small. Patients on anticoagulants were included in the trial based on clinician discretion, which may have biased towards inclusion of overall lower risk patients. Furthermore, anticoagulant use was associated with increased risk of macrohemorrhages in patients treated with lecanemab, though numbers were also small [7]. Given the high morbidity and mortality associated with macrohemorrhages on a background of anticoagulation [67], the Work Group recommends *against* use of donanemab in patients on anticoagulants. This recommendation may change as more safety data emerge from additional clinical trials and longitudinal clinical registries following treated patients.

### 5.3. Differential diagnosis of ARIA vs. stroke and use of thrombolytics

Symptomatic ARIA can rarely present with focal neurologic deficits that mimic acute stroke. Differentiating ARIA from stroke in the emergency department setting is critically important, since use of intravenous thrombolytics in patients being treated with donanemab and other A*β*-targeting monoclonal antibodies has been associated with severe outcomes, including death [3,48,49]. Fatal bilateral intracerebral hemorrhages occurred shortly after intravenous tenecteplase in a 72-year-old donanemab-treated man, heterozygous for *APOE4*,who presented with headache and slurred speech 7 days after a fifth dose in the long-term addendum [71]. Pre-thrombolysis CT angiogram and perfusion studies in this patient were reportedly negative for vessel occlusion or other acute changes, whereas post-thrombolytic MRI demonstrated severe ARIA-E, superficial siderosis, and multifocal lobar hemorrhages. A patient heterozygous for *APOE4* in TRAILBLAZER6 also had a fatal hemorrhage 2 days after receiving tenecteplase for presumed acute stroke. The patient presented with hemiparesis and seizures, and showed hypodensity in the right parietal lobe on CT, though retrospectively this imaging finding represented ARIA-E [61]. Similarly, a fatal case of hemorrhage occurred in a patient homozygous for *APOE4* following alteplase thrombolysis in the open-label phase of the lecanemab CLARITY trial. The neuropathology in that case revealed multifocal intracerebral hemorrhages and severe cerebral amyloid angiopathy-related inflammation [49]. The FDA prescribing information for donanemab includes a boxed warning that “treating clinicians should consider whether [focal neurologic] symptoms could be due to ARIA-E before giving thrombolytic therapy” [4]. Based on these reports, the Work Group recommends that intravenous thrombolysis not be administered to patients receiving donanemab without a pre-treatment MRI unequivocally excluding ARIA. Notably, a “stroke protocol” head CT/CT angiogram may fail to detect even advanced ARIA. Conversely, severe cases of ARIA-E can be associated with punctate regions of restricted diffusion on DWI consistent with acute ischemia, but these are typically much smaller than the extensive regions of edema and hemorrhage. Mechanical thrombectomy performed without a thrombolytic does not appear to cause CAA-related intracerebral hemorrhage [72] and should be considered for acute stroke due to large-vessel occlusion in donanemab-treated patients.

### 5.4. Management of ARIA

**Fig. 2.**
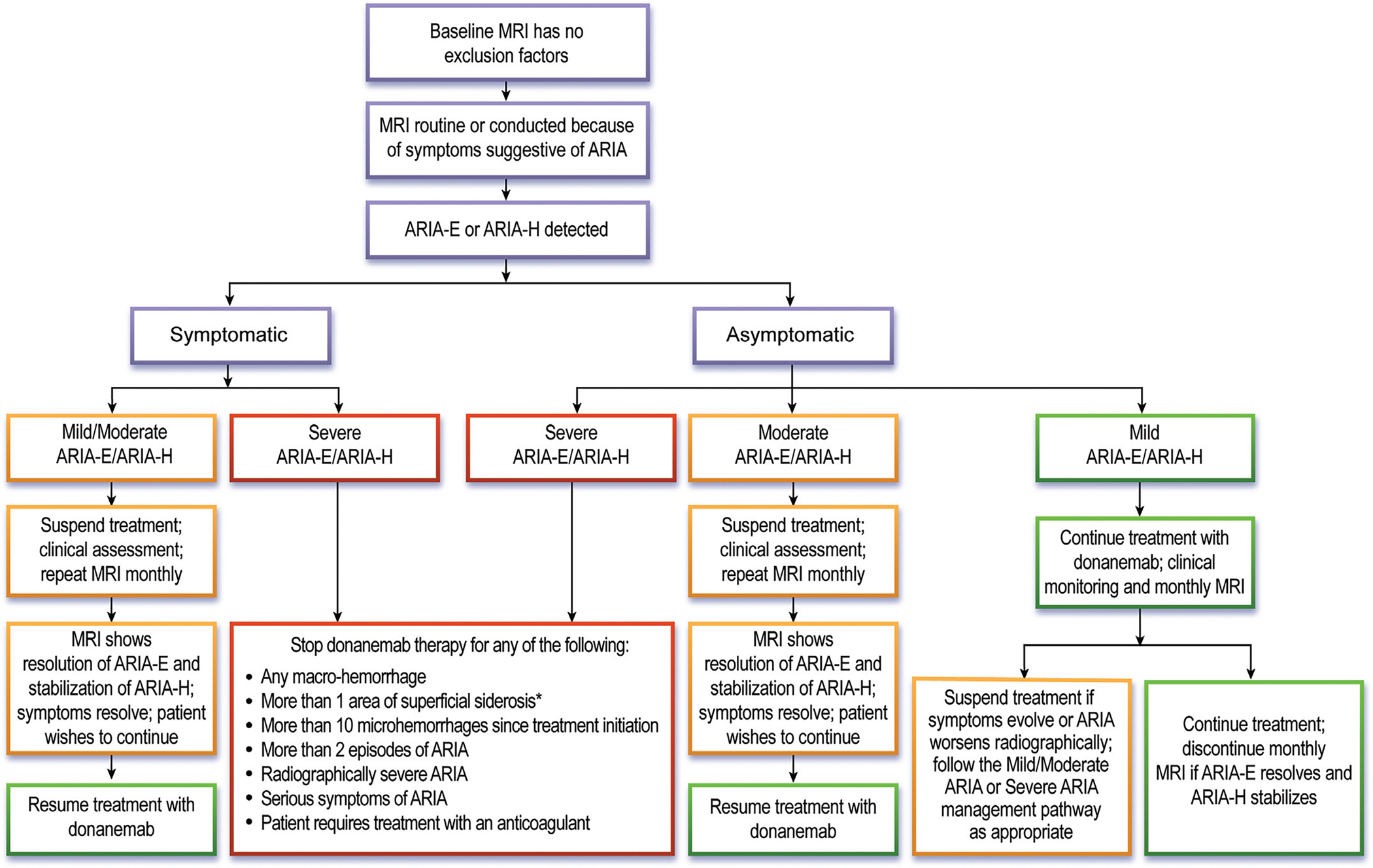
Monitoring and management of ARIA. Flow chart lists recommended actions based on classification of ARIA as symptomatic/asymptomatic and based on the grading of ARIA radiographic severity. Action boxes are color coded: green – continue treatment; orange – suspend treatment; red – stop treatment. * - We recommend discussing the risks/benefits of continued treatment with the patient and care partners if a single area of cortical superficial siderosis emerges during treatment.

The management of ARIA is based on the radiographic severity (Table 7) and presence or absence of symptoms (Fig. 2, Table 8). Donanemab treatment should be discontinued if patients show radiographically severe ARIA-E or ARIA-H, regardless of the presence or absence of symptoms. If ARIA is symptomatic or radiographically rated as moderate, donanemab dosing should be suspended. Patients should be monitored with monthly MRI scans, and treatment can be resumed if symptoms have fully resolved, ARIA-E has resolved, ARIA-H has stabilized, stopping rules have not been met (see below), and the patient and care partners wish to continue treatment. In patients with radiographically mild and asymptomatic ARIA, donanemab dosing can be continued, with vigilant monitoring for symptoms and monthly MRI scans to evaluate the evolution of the radiographic changes. If the patient remains asymptomatic, ARIA-E resolves and ARIA-H stabilizes, monthly MRI scans can be discontinued, and MRI monitoring can return to the surveillance schedule (Fig. 1A, B). If radiographic ARIA worsens to moderate or the patient becomes symptomatic, donanemab dosing should be suspended, the patient should be clinically monitored closely, and monthly MRIs should be continued. These recommendations are identical to those listed in the lecanemab AUR [7], and largely align with the FDA donanemab prescribing information [4], although the latter allows for continuing treatment in patients with mild symptoms and radiographically mild ARIA-E based on clinician judgment. While we recommend a slightly more conservative approach, we emphasize that clinical judgment is paramount, and prescribers should consider additional patient factors (e.g., nature and severity of symptoms, ARIA history and evolution, comorbidities, concurrent medications, *APOE* genotype) in managing ARIA. Given the importance of accurately detecting and rating ARIA, the American Society of Neuroradiology’s “Alzheimer’s, ARIA and Dementia Study Group” has issued updated recommendations for standardized MRI protocols, workflows, and reporting on imaging studies obtained to assess ARIA [54]. Several companies are developing automated software packages that utilize artificial intelligence to detect and rate ARIA and help supplement the radiologist’s visual interpretation.

### 5.5. Management of serious and severe ARIA

1.5 % of ARIA-E and 0.4 % of ARIA-H cases in the donanemab placebo-controlled trials were considered serious, including 3 ARIA-related deaths in 984 participants [48]. Rates of serious ARIA-E and ARIA-H were 0.7 % and 0.3 % respectively in the open-label addendum, with two ARIA-related deaths reported in 1047 participants and an additional ARIA-related death in the TRAILBLAZER-ALZ6 trial. Accordingly, clinicians and health systems that prescribe donanemab need to have the appropriate care pathways and resources in place to screen, detect, monitor, mitigate risk of complications, and manage ARIA, including assessment and management of suspected serious or severe ARIA (Table 9). Severe cases of ARIA typically present with the radiographic syndrome of CAA-ri [52], with large areas of edema (with or without mass effect) noted on MRI. ARIA-E and ARIA-H can co-occur in the same regions, with multiple microbleeds noted in the regions of most severe edema. Diffusion weighted imaging can reveal punctate areas of restricted diffusion, but these are typically minor compared to the large regions of edema or hemorrhage.

Most serious and severe ARIA cases occur within the first three months of initiating treatment with donanemab. The incidence of severe ARIA was partially mitigated by modification of the TRAILBLAZER-ALZ2 protocol to include an MRI at week 4, and this is now recommended as standard practice in the AUR and the prescribing information (Fig. 1A, B). Overall, radiographically severe ARIA is more likely to be associated with symptoms, though, at an individual level, patients with severe radiographic ARIA can range from being asymptomatic to experiencing serious neurological symptoms (Table 5). In addition to misdiagnosis as stroke, severe ARIA may also be mistaken with posterior reversible encephalopathy syndrome [73] given overlapping clinical features and radiographic findings.

Patients with serious or severe ARIA may require admission to the hospital or intensive care unit (ICU) for stabilization, monitoring and treatment. Based on the totality of evidence, our experience managing severe cases of CAA-ri and managing ARIA-E in clinical trials, we recommend considering early initiation of high-dose glucocorticoid treatment (e.g., methylprednisolone 1*g* intravenously per day for 5 days followed by an oral steroid taper over several weeks) [74]. Patients should be monitored for neurological status, seizures and other potential complications. Outcomes of serious and severe ARIA have ranged from, most commonly, complete clinical recovery to, occasionally, permanent disability and, rarely, to death. It is important that clinicians appropriately discuss these possibilities with patients and care partners before initiating treatment, and also as part of ongoing discussions, especially when ARIA or other treatment-related complications arise.

### 5.6. Stopping treatment due to ARIA

ARIA-based stopping criteria are shown in Fig. 2. We recommend discontinuing donanemab if patients develop radiographically severe ARIA-E or ARIA-H, any macrohemorrhage, more than 10 new microhemorrhages since initiating treatment, or more than one area of superficial siderosis. Given the emerging potential association between baseline cSS and serious ARIA, we recommend re-evaluating the risks and benefits of continuing treatment even if only a single area of siderosis emerges. We also recommend discontinuing treatment in patients who develop a third ARIA event (even if mild and asymptomatic), those with a seriously symptomatic episode of ARIA and in patients who develop a new medical indication for treatment with an anticoagulant.

## 6. Infusion reactions

In TRAILBLAZER-ALZ2, infusion-related reactions occurred in 8.7 % of patients treated with donanemab, compared to 0.5 % of patients receiving placebo [3]. Reactions typically developed within 30 min of initiating the infusions, and 70 % occurred within the first 4 infusions. Common symptoms of infusion-related reactions included fever and flu-like symptoms (chills, generalized aches, feeling shaky, and joint pain), nausea, vomiting, hypotension, hypertension, and oxygen desaturation. Reactions are graded based on the severity and duration of the symptoms (Table 10) [75,76]. Most infusion-related reactions are self-limited and can be managed safely at home. In the phase 3 clinical trial, 57 % of reactions were graded as mild and 39 % as moderate.

Donanemab infusions should be stopped immediately if patients are experiencing grade 2 or higher infusion reactions. Mild symptoms are treated with diphenhydramine and acetaminophen every 4–6 h until symptoms resolve. If the patient has been discharged from the infusion center, a call should be placed to the patient or care partner to assess whether symptoms have resolved. More significant or persistent symptoms are treated with oral dexamethasone (0.75 mg/ day for 2–3 days) or oral methylprednisolone (80 mg twice per day for 2–3 days). Diphenhydramine or topical corticosteroid creams can be prescribed to treat skin hypersensitivity reactions. Antihypertensive treatment may be needed if patients develop significant blood pressure elevations. Clinics should be prepared with bronchodilators, oxygen, epinephrine, and intravenous steroids for severe infusion-related reactions, which are exceedingly rare but potentially life-threatening.

Donanemab treatment should be discontinued if patients experience grade 3 or higher infusion-related reactions. For patients who experienced a grade 1–2 infusion-related reaction in their previous dose, prophylactic pre-treatment should be initiated with diphenhydramine 25–50 mg and acetaminophen 650 mg-1000 mg 30 min prior to the next infusion. If this proves ineffective, prophylactic treatment can be escalated to oral dexamethasone (0.75 mg) or oral methylprednisolone (80 mg) 6 h before infusion. The prophylactic regimen should be maintained until the patient remains asymptomatic following 2–4 further infusions.

## 7. Amyloid PET based discontinuation of treatment

A unique aspect of TRAILBLAZER-ALZ and TRAILBLAZER-ALZ2 is that treatment duration was based on amyloid plaque clearance [2,3]. Amyloid PET scans were performed every 6 months, and patients randomized to active treatment were switched to placebo if PET-based stopping criteria were met (single amyloid PET measurement less than 11 CL, or two consecutive measurements greater than 11 CL and less than 25 CL). The rationale for limiting the duration of treatment is grounded in the selectivity of donanemab for N3PE-A*β* [1] which is present in the insoluble, mature plaques imaged by PET. If the specific A*β* antigen targeted is assumed to be quantitatively reduced or virtually eliminated by donanemab treatment, there is little theoretical justification for continued treatment. Stopping donanemab dosing could also limit the impact of anti-drug antibodies, which developed in > 80 % of donanemab clinical trial participants.

The clinical safety and efficacy of donanemab was established based on limited duration dosing. In the combined tau population in TRAILBLAZER-ALZ2, amyloid clearance was achieved in 29.7 % of participants at 24 weeks, 66 % of participants at 52 weeks, and 76.4 % at 76 weeks. Moreover, average amyloid PET quantification in individuals who met stopping criteria was approximately 0 CL, with subsequent accumulation rates of 2.8 CL/year on follow-up PET scans, suggesting individuals would potentially remain below a typical threshold for amyloid positivity (~25 CL [30]) for years to come.

Counter-balancing the rationale for discontinuing treatment based on amyloid PET in clinical practice are both scientific and pragmatic concerns. From a scientific perspective, pivotal trials of earlier amyloid-lowering antibodies (e.g., aducanumab, lecanemab) did not terminate antibody dosing when specific levels of amyloid clearance were reached. Notably, these antibodies target A*β* protofibrils that represent an earlier stage of the A*β* aggregation cascade compared to the N3PE epitope on mature plaques targeted by donanemab. In the open-label extension of the Phase 2 lecanemab study, plasma A*β*42/40 ratio and p-tau181 levels began to return toward abnormal pre-randomization levels during the “gap phase” in which patients were not receiving active treatment, suggesting that the AD pathological cascade may resume shortly after discontinuing A*β*-targeting antibodies [77]. From a pragmatic point of view, financial coverage of multiple amyloid PET scans by payers is currently unclear, and scan interpretation in clinical practice does not routinely include the quantification used to implement stopping rules in the TRAILBLAZER trials. Moreover, the methodology for clinical visual reads has not yet been validated for establishing treatment-related amyloid clearance, and amyloid PET scans of patients undergoing A*β* lowering therapies treatment may differ qualitatively from those of treatment-naïve individuals. Plasma A*β* or p-tau levels do not correspond well enough with lowered plaque levels shown by amyloid PET to be used to guide stopping therapy.

Overall, the Work Group acknowledged that there are gaps in our understanding of the optimal duration of donanemab treatment, as well as the ability to implement amyloid PET based stopping rules in clinical practice. Given the design of the TRAILBLAZER study, the biological target of donanemab and the development of anti-drug antibodies with extended therapy, the Work Group concluded that it is reasonable for providers to consider stopping therapy based on a follow-up amyloid PET scan, typically obtained 12–18 months after initiating treatment, is read as negative. This recommendation assumes that longitudinal amyloid PET can be obtained and interpreted reliably in clinical practice. Clinicians may consider pre-treatment amyloid PET quantification (if available) and the mean rate of amyloid clearance in the clinical trial (~60 CL/year) in determining the optimal timing for a repeat PET scan to assess for amyloid clearance. In settings where amyloid PET is not available to monitor treatment response, clinicians may choose to limit the duration of treatment, for example limit treatment to 18 months based on the knowledge that over 75 % of patients in TRAILBLAZER-ALZ2 achieved amyloid clearance on PET in that timeframe.

## 8. Switching to donanemab from another A*β*-targeting monoclonal antibody

With three A*β*-targeting antibodies currently approved by the FDA, we anticipate that patients or providers may seek to transition treatment from one approved drug to another in class. There are no clinical trials addressing switching between A*β*-targeting antibodies, and no evidence-based guidelines for switching based on efficacy or safety. Pragmatic reasons may arise – for example, production of aducanumab has been stopped, and patients on aducanumab treatment may elect to switch to another approved antibody.

We recommend that patients be off the previous agent for 5 half-lives prior to the initiation of the alternative antibody. At the end of five half-lives, the level of the original agent will be reduced by approximately 97.5 %; this figure is subject to influence by a variety of host factors [78]. The half-life of aducanumab is 24.3 days [79]; the half-life of lecanemab is 9.5 days [80]; and the half-life of donanemab is 11.8 days [81]. Therefore, patients should be off aducanumab for approximately 4 months or off lecanemab or donanemab for approximately 6 weeks prior to initiation of a different anti-amyloid monoclonal antibody. If patients are switched to donanemab from another anti-amyloid monoclonal antibody, donanemab should be titrated as when initiating therapy in a treatment-naïve patient (Fig. 1A,B).

Prior to switching, a repeat amyloid PET should be obtained, and if plaque burden as assessed by PET has been reduced to below detectable levels by therapy with a previous antibody (“treatment-related amyloid clearance”), treatment with donanemab should not be initiated. If amyloid PET remains positive, a new baseline brain MRI should be obtained prior to starting donanemab, and patients should not be treated if they meet MRI exclusion criteria (Table 2). Similarly, patients should not be switched from one monoclonal antibody to another if they meet any of the other safety stopping rules (Fig. 2).

## 9. Patient-centered, supported, and shared decision-making processes

A central and necessary core principle of good clinical practice is autonomy and respect for persons. In the context of AD care in general, the goal is to achieve and maintain a well-informed and well-advised person-centered and personalized process that empowers the patient-care partner dyad to maximally exercise autonomy via supported and shared decision-making. To achieve this, individualized, thoughtful, honest, compassionate and open communication is needed. For the person being considered for donanemab therapy, a triadic relationship between the clinical team and the patient-care partner dyad is maintained throughout the duration of care. Promoting well-informed shared decision-making is a dynamic process that utilizes both the art and science of medicine, and is maintained throughout consideration and assessments for treatment candidacy; initiation, maintenance and monitoring of treatment; and during any potential modification, treatment pause or discontinuation, or monitoring and management of side-effects or complications. The Work Group recognizes that clinicians will be challenged not only to effectively communicate and navigate a rapidly evolving and new paradigm of AD care, with complicated biopsychosocial and environment considerations, but to do so within the inter-related complexities impacting the patient-care partner dyad, and the clinician, clinical team, health system and payers, that currently involve implementation gaps and constraints in access, coverage, costs, logistics, readiness and resources.

Continued education on the AD disease state, treatment and care options are foundational in this process. This includes educating dyads regarding current terminology, definitions, understandings - and the pathobiological and clinical spectrum and staging of AD; and uncertainties, unknowns and nuances regarding AD diagnosis, treatments and prognosis. The rationale for AD treatment and care strategies, including alternatives to A*β* plaque-lowering monoclonal antibody therapies; non-pharmacological, pharmacological, and lifestyle approaches [82]; conceptual differences between symptomatic and disease-modifying therapies; and detection and management of co-morbid and potentially contributing conditions should all be explained. It is important to clearly explain nuances regarding extrapolation of clinical trial population-based averages and expectations to individual person-based clinical care; the timeframes (e.g., 18 months) and populations tested in clinical trials; and the uncertainties regarding longer-term treatment.

Clinicians should provide a realistic description of the goals of treatment, i.e., removal of A*β* plaques and slowing of clinical decline. Patients and care partners should be fully aware that treatment is not a cure, is not expected to improve clinical symptoms, is unlikely to completely arrest disease progression, and will not, over the long-term, prevent progression of symptoms and functional and clinical decline. Expected benefits should be framed in terms that are both understandable and meaningful to patients and care partners (e.g., delaying progression in global disease stage, “extended time” in clinically mild stages, “time saved” to do enjoyable activities, preservation of independence in functional capacities) [83,84]. If patients fall outside the population included in the clinical trials (e.g., younger or older age, atypical AD phenotype), clinicians should be transparent in presenting uncertainties regarding clinical benefit. Additionally, the donanemab clinical trials had limited ethnoracial diversity, with 6.0 % of all participants identifying as Asian, 2.3 % of all participants identifying as Black or African-American, and 5.7 % of U.S. participants identifying as Hispanic/Latino [3]. Given known disparities across ethnoracial groups in rates of amyloid positivity, prevalence of comorbidities, other dementia risk factors as well as genetic associations with AD [85–88], it is possible that treatment-related benefits and risks may differ by ethnoracial group. Clinicians should be transparent about these uncertainties when counseling patients from minoritized populations that were under-represented in the donanemab clinical trials.

Counter-balancing anticipated benefits, supported and shared decision making should involve a detailed discussion of treatment risks, including risks of ARIA (based on patient’s *APOE* genotype and other risk factors) and the rare but potentially disabling and life-threatening risk of serious ARIA. Patients should be counseled about medications they will need to avoid while on treatment (e.g., anticoagulants and thrombolytics), the requirement for prompt communication of any new symptoms, medical conditions or medication changes; and that their treatment options may be limited should they develop a condition that could otherwise optimally require acute anticoagulation or thrombolysis, including a potential stroke. Treatment burden on patients should also be discussed, including the commitment to monthly infusions, biomarker testing via PET or CSF, *APOE* genotyping (and its ramifications for first degree family members) and frequent MRIs performed for ARIA monitoring early in the treatment course. Any potential co-pays and out-of-pocket expenses related to the treatment and ancillary tests and procedures should be clarified. Finally, alternative treatment approaches, including use of symptomatic therapies, lifestyle modifications and participation in clinical trials should be discussed. Patients should be aware that by receiving treatment they may lose eligibility to participate in clinical trials, since most current trials for early symptomatic AD exclude patients receiving A*β*-targeting therapies as part of their clinical care (though this is expected to change in the future as combination therapy trials become more common) [89].

Patients with early symptomatic AD may have retained medical decision-making capacity, yet still be challenged to process the nuanced risk-benefit trade-offs associated with donanemab treatment [90]. Patient-friendly visualization and decision-making tools may be useful aids for patients and care partners in this process [91]. The institutions of some Work Group members have developed AD monoclonal antibody care pathways with rigorous clinical workflows, templates, processes and protocols, including requirement for formalized clinician attestations and patient-care partners consent processes for prescribing, administering and managing donanemab and lecanemab. Even in instances when formal consent documentation is not required, clinicians should carefully assess and document the patient-care partner dyad’s capacity to appreciate the benefits and risks of treatment and make an informed decision that aligns with their values and goals; and that they have the ability and have agreed to appropriately participate in their donanemab treatment and care requirements. If a patient is deemed to lack capacity, a legally authorized representative should be the primary decision maker, and patient assent should be confirmed for initiating and continuing treatment.

## 10. Clinical resources and workflow

Safe and effective use of donanemab and other A*β*-targeting antibodies requires significant clinical resources and multidisciplinary expertise (Table 11). Prescribing clinicians must have expertise in the evaluation and differential diagnosis of early-stage cognitive-behavioral impairment, access to AD biomarker testing (CSF or PET) and *APOE* genotyping, and the clinical proficiency to interpret genetic and biomarker results and assess brain MRIs across a wide array of clinical scenarios. Patients and clinicians must have access to infusion centers that can accommodate monthly infusions and safely manage infusion reactions. Prescribers and patients require access to radiology services with the capacity to perform baseline (and potentially follow-up) amyloid PET scans or baseline lumbar punctures, adhere to the MRI monitoring schedule, and provide and appropriately communicate clinical reads by imaging specialists who are trained in amyloid PET interpretation and ARIA detection. Close coordination with emergency department, inpatient and intensive care unit (ICU) services is needed to safely assess cases of suspected ARIA and manage severe and serious ARIA across its clinical manifestations and complications.

In nearly all practice settings, administration of donanemab and other drugs-in-class will require the development of new and more resource-intensive clinical workflows compared to the previous standard-of-care for AD and related disorders. Yet this challenge also represents an opportunity to globally elevate AD care, for example by enabling widespread implementation of AD biomarkers in clinical practice, thus enhancing access to a timely and accurate diagnosis, which will have broader benefits beyond patients who ultimately elect to undergo donanemab treatment. A*β*-targeting monoclonal antibody care pathways and clinical workflows may include treatment referral review by an internal multidisciplinary expert group or committee (akin to a tumor board review); regular coordination and training of staff; a patient wallet ID with dyad with contact information of treating team and instructions and cautions related to ARIA (e.g., symptoms) and donanemab treatment (e.g., contra-indication to anticoagulants and thrombolytics); electronic health record (EHR) alerts and flagging for patients on donanemab treatment; and EHR order sets and templates for donanemab prescription and infusions (e.g., ARIA symptoms checklist prior to infusions; medication orders for infusion-related reaction treatment and prophylaxis), as well as for MRIs (e.g., appropriate sequences, template ARIA assessment reports).

## 11. Discussion

The AUR described in this manuscript are intended to provide an independent, academic perspective on the safe and effective translation of donanemab into real-world clinical practice. As a newly approved drug, donanemab’s safety and efficacy have thus far been characterized primarily in highly controlled clinical trial settings [2,3]. Compared to clinical trial participants, real-world patients with early symptomatic AD are older, less educated, more ethnoracially diverse, have a higher burden of medical comorbidities, and receive more concomitant medications [8]. Real-world patients are treated by clinicians who have less experience with this novel class of drugs, and do not have access to the central safety review and monitoring guardrails available in the clinical trials setting. The goal of the AUR is to assist clinicians who are considering prescribing donanemab to appropriately select patients who are most similar to those included in the clinical trials and thus have the best-established safety and efficacy data. Furthermore, we seek to guide clinicians in safe implementation of the drug by recommending ARIA monitoring and management strategies that have proven effective in clinical trials [2,3].

While the AUR are largely aligned with the TRAILBLAZER-ALZ2 clinical protocol and FDA prescribing information, in some instances, we have taken a more conservative position on exclusion criteria, ARIA management and drug discontinuation. For example, we recommend excluding patients on chronic anticoagulation, even though a limited number of these patients were included in the clinical trial. Our rationale for making more conservative recommendations is to prioritize patient safety in the early roll-out of donanemab, given the very rare but potentially life-threatening complication of serious and severe ARIA [48]. This is especially true as the drug is initially being prescribed to a broader patient population, and in healthcare settings with limited experience with A*β*-targeting antibodies and their potential complications. In some instances, we recommend more conservative criteria based on learnings from the donanemab clinical trials and trials of other drugs-in-class. For example, we recommend excluding patients with baseline cSS based on a growing number of cases of serious ARIA with poor outcomes, including intracerebral hemorrhages and deaths, that have occurred in this group of patients [3,55]. We recommend suspending treatment in patients with symptomatic ARIA, even if symptoms are mild. This recommendation is based on cases in which mild symptomatic ARIA evolved into severe ARIA with continued treatment. In other instances, the AUR are more permissive than the clinical trial criteria, in order to make practical accommodations for real-world clinical practice and application to diverse (medically and phenotypically) real-world patient populations. For example, we do not recommend excluding patients under age 60 or over age 85, those with non-amnestic AD phenotypes, or patients with ADAD mutations (aside from patients with mutations that are specifically associated with CAA), and we do not require tau PET or quantitative amyloid PET thresholds for treatment. We continue to restrict treatment initiation to patients in AD Clinical Stages 3–4 (MCI or mild dementia), pending the results of ongoing clinical trials in earlier, preclinical stages of AD, in which A*β* lowering may be associated with even greater clinical benefit. As suggested by their name, the AUR are intended as *recommendations*,not criteria, guidelines or requirements. They are not intended to supplant clinical judgment, as we recognize that no set of recommendations can fully anticipate or reproduce the complexities of clinical practice. Furthermore, we anticipate our recommendations will likely evolve as more real-world data are accrued. In this spirit, we strongly encourage prescribing clinicians to enroll patients treated with donanemab in the Alzheimer’s Network (ALZNET; https://www.alz-net.org) or similar real-world registries designed to accrue longitudinal safety and effectiveness data on patients treated with novel AD therapies.

Several innovative aspects of the TRAILBLAZER-ALZ and TRAILBLAZER-ALZ2 trial design advanced our understanding of precision medicine approaches to AD therapy yet may be challenging to replicate in current clinical practice [2,3]. In the clinical trials, baseline tau PET proved to be a strong predictor of clinical response, with patients with low-medium tau showing greater clinical benefit than those with baseline high tau burden [3]. The important role of tau PET in biological staging of AD is acknowledged in the revised Alzheimer’s Association criteria for diagnosis and staging of AD [9]. However, tau PET is not currently available in most clinical care settings, and clinical interpretations are based on a binary visual read which does not stratify patients based on tau PET burden [46]. Based on these limitations, the AUR do not require tau PET for determining treatment eligibility but acknowledge that tau PET could be used to individualize the estimate of clinical response at centers that are equipped to acquire and interpret the scans accordingly. In our experience tau PET may be particularly useful in cases that are deemed “borderline” by other criteria – for example in patients on the lower end of the acceptable MMSE range, or in patients at higher risk of ARIA based on *APOE* genotype or baseline MRI findings. In these select patients, a lower tau PET burden may tip the overall risk-benefit calculation in favor of treatment, whereas a higher tau PET burden may tip the balance against treatment.

Another novel aspect of the donanemab clinical trials design was limiting the duration of treatment based on amyloid PET response [2,3]. Overall, the Work Group identified a strong biological rationale for this approach, as well as potential benefits for patients, including limiting the burden of treatment, the duration of exposure to ARIA risk, and the potential impact of anti-drug antibodies. Counter-balancing these arguments, we identified an absence of comparative randomized data on continuing treatment, challenges around access to, and interpretation of, longitudinal amyloid PET in clinical practice, and the possible use of donanemab to prevent the recurrence of the N3PE-A*β*. Further, we do not know if fluid biomarkers measured in CSF or blood could be applied to determine amyloid clearance in patients with limited access to PET. On balance, we recommend considering discontinuing donanemab if repeat amyloid PET, typically obtained 12–18 months after initiating therapy, is available and read as negative.

Our recommendations are aligned with updated Appropriate Use Criteria for amyloid and tau PET, which identify a clinical role for amyloid and tau PET in determining eligibility for A*β*-targeting therapies, and a clinical role for amyloid PET in monitoring treatment response [92]. With their growing role in clinical care, we believe both amyloid and tau PET would benefit from incorporation of image quantification into clinical interpretations, to enhance diagnostic accuracy, facilitate longitudinal measurements, and optimize the potential of molecular imaging to guide management decisions in AD. The Work Group also recognizes the potential of blood AD biomarkers to enhance access to, and afford-ability of, AD biomarker testing. Though the current iteration of the AUR requires PET or CSF confirmation of AD pathology, we anticipate that several blood tests will be shown to meet the high standards for diagnostic performance required for regulatory approval. If a specific blood test is cleared by the FDA, shows equivalent diagnostic accuracy to CSF or PET and meets the performance requirements outlined in a recent consensus paper, the Work Group would deem that test to be appropriate for determination of treatment eligibility for A*β*-targeting therapies [37].

The recommendations presented in the AUR for stopping treatment are grounded in severe adverse events (i.e., severe ARIA or infusion reactions) or in amyloid-PET response. As noted earlier, in practice settings in which amyloid PET is not available to gauge treatment response, it may be reasonable to discontinue the drug after 76 weeks (18 months) given that > 75 % of patients in TRAILBLAZER-ALZ2 achieved amyloid clearance following this duration of treatment [3]. Aside from these considerations, there is currently a paucity of data to guide clinicians about the optimal duration of donanemab treatment. Clinical progression is expected in treated patients, and the decision to continue or stop treatment in the face of disease progression should be grounded in a continuous shared decision-making process that weighs the evolving dynamics of risks vs. benefits. Based on both the drug’s mechanism-of-action and empiric data, it is expected that patients in earlier clinical stages of AD will derive greater clinical benefit. Conversely, once a patient progresses into the moderate stages of dementia (equivalent to Global CDR = 2 or Clinical Stage 5), in which they are experiencing severe impairment in memory and other cognitive functions and requiring assistance with basic activities of daily living, it is reasonable to re-evaluate whether continued treatment is warranted given possibly diminishing clinical benefits. Future data from open-label extension studies and real-world registries are needed to further address this significant gap in knowledge, which is highly relevant to clinical implementation of donanemab and other A*β*-targeting antibodies.

Donanemab joins lecanemab as the second A*β*-targeting antibody to receive full FDA approval based on evidence of clinical efficacy in placebo-controlled randomized trials [3,63]. Clinicians and patients now have a choice between two A*β* lowering therapies for early clinical stages of AD. It is important to recognize that the two pivotal phase 3 trials, CLARITY-AD (lecanemab) and TRAILBLAZER-ALZ2 (donanemab,) employed different biomarker and clinical inclusion/exclusion criteria, resulting in subtle but potentially meaningful differences in patient cohorts. Specifically, patients in TRAILBLAZER-ALZ2 were more clinically and biologically advanced than those in CLARITY-AD [3,63]. Therefore, direct comparisons of safety and efficacy data between the two trials are not valid. Keeping that important caveat in mind, observed rates of ARIA in the phase 3 trials were higher with donanemab than lecanemab [3,63]. This difference may in part be explained by more advanced amyloid pathology in the TRAILBLAZER-ALZ2 study and may in the future prove to be mitigated by the alternative donanemab titration schedule (Fig. 1 B). Nevertheless, for the time being it may be prudent to consider this factor when choosing a drug for patients at highest risk of ARIA. Another difference to consider is the potential for limited duration of donanemab treatment (based on amyloid PET response), versus the more open-ended duration of lecanemab treatment, including a lower frequency maintenance dosing schedule following 18 months of bi-weekly treatment recently approved by the FDA. Ultimately, in lieu of a head-to-head randomized clinical trial, one cannot make evidence-based recommendations for choosing between the two approved and available antibodies. Treatment decisions for individual patients will likely often be grounded in pragmatic considerations, e.g., scheduling of infusions, safety MRIs, payer coverage, etc. It is also likely that patients will elect to switch from one antibody to another based on tolerability or perceived efficacy.

## Summary

The AUR integrate data from the donanemab clinical trials to assist practitioners who have decided to use the drug in identifying appropriate patients and safely administering treatment in the clinical setting. We recommend that clinical use of donanemab be applied to a similar patient population to that included in the clinical trials, i.e., patients with MCI or mild dementia due to AD (Clinical Stages 3–4) who have established AD pathology confirmed by amyloid PET or CSF tests. Tau PET is not required to determine eligibility but can be used when available to individualize estimates of treatment benefit. Patients with pre-treatment MRI findings of >4 microbleeds, any cSS, or a major vascular contribution to cognitive impairment should be excluded from treatment, as should patients with clinical or imaging findings suggestive of an alternative (non-AD) cause of cognitive impairment, and patients requiring chronic anti-coagulation. *APOE* genotyping should be performed prior to treatment to inform an individual’s risk of developing ARIA. The decision to initiate therapy should be grounded in a shared decision-making process that emphasizes the patient’s values and goals of care. Donanemab is administered as a monthly intravenous infusion, with escalating dose titration when initiating treatment. MRI scans to evaluate for ARIA should be routinely performed prior to the 2nd, 3rd, 4th and 7th infusions, and at any time ARIA is suspected clinically. An additional MRI prior to the 12th infusion can be considered in patients at highest risk for ARIA. Clinicians may consider discontinuing treatment if treatment-related amyloid clearance is demonstrated by amyloid PET, typically performed 12–18 months after initiating treatment.

## Figures and Tables

**Table 1:** Diagnostic criteria for mild cognitive impairment (MCI) and dementia with mild functional impairment, adapted from [9,10]. Positive Core 1 biomarkers are needed to establish AD as the etiologic cause of cognitive impairment.

Syndrome	Definition

Mild cognitive impairment (MCI) / Clinical Stage 3	• Cognitive concerns by the patient, knowledgeable informant, or the physician • Objective impairment in one or more cognitive domains including memory, executive function, attention, language, or visuospatial skills • Generally preserved activities of daily living (ADL) • No dementia
Dementia with mild functional impairment / Clinical Stage 4	• Cognitive concerns by the patient, knowledgeable informant, or the physician • Performance in the impaired/abnormal range on objective cognitive tests • Evidence of decline from baseline, documented by the individual’s report or by observer (e.g. study partner) report or by change on longitudinal cognitive testing or neurobehavioral behavioral assessments • Progressive cognitive and mild functional impairment on instrumental ADL with independence in basic ADL

**Table 2:** Eligibility criteria used in the TRAILBLAZER-ALZ2 Phase 3 trial of donanemab and corresponding Appropriate Use Recommendations (AURs).

Eligibility Criteria Applied in the TRAILBLAZER-ALZ2 Phase 3 Trial of Donanemab	Appropriate Use Recommendations for Patients Considered for Treatment with Donanemab

Inclusion Criteria	
60–85 years of age	Physician judgement used for patients outside the 60–85 year age range
Early symptomatic AD (MCI [10] or AD with mild dementia [25]) with gradual and progressive change in memory function reported by the participant or informant for ≥ 6 months	Clinical diagnosis of MCI or dementia with mild functional impairment as defined in Table 1, with AD as the suspected etiology based on cognitive-behavioral syndrome
MMSE scores of 20-28	MMSE 20-30, MoCA 13-30, or other cognitive screening instrument with a score compatible with early AD. Clinician judgment is warranted where specific patient related circumstances may affect MMSE or MoCA performance, such as low education or lack of proficiency in the language of the test
Positive amyloid PET (^18^F-florbetapir or ^18^F-florbetaben), operationalized by central image evaluation and quantification of global amyloid PET uptake ≥ 37 Centiloids	Positive amyloid PET based on clinical read, or CSF studies indicative of AD pathology (e.g., A*β*42/A*β*40 ratio, or p-tau181/A*β*42 ratio)
Tau pathology assessed by 18F-flortaucipir imaging with central image evaluation and quantification	Tau PET is not required for donanemab use, but if available can be used to individualize the estimate of clinical benefit
Stable concomitant symptomatic AD medications and other medications that may impact cognition for at least 30 days prior to randomization (does not apply to topical, as needed [prn], or discontinued medications)	May be on cognitive enhancing agents (donepezil, rivastigmine, galantamine, or memantine) for AD; patients may not currently be on aducanumab or lecanemab; patients may be on standard of care for other medical illnesses
Contraceptive use by men or women should be consistent with local regulations regarding the methods of contraception for those participating in clinical studies	Treated patients should understand that the effect of donanemab on the ability to have children or its effect on the unborn fetus are unknown
Study partner who will provide written informed consent to participate, is in frequent contact with the participant (defined as at least 10 h per week), and will accompany the participant to study visits or be available by telephone at designated times	Care partner or family member(s) who can ensure that the patient has the support needed to be treated with donanemab
Adequate literacy, vision, and hearing for neuropsychological testing in the opinion of the investigator at the time of screening	In the opinion of the clinician, have adequate literacy, vision, and hearing for cognitive testing
Participants are reliable and willing to make themselves available for the duration of the study and are willing to follow study procedures	Treated patients are reliable and are willing to follow study procedures
Participants or legally authorized representatives capable of giving signed informed consent which includes compliance with the requirements and restrictions listed in the informed consent form and in the protocol	Patients, care partners, and appropriate family members understand the requirements for donanemab therapy and the potential benefits and harms of treatment. Approval by surrogate decision maker with patient assent may be appropriate if patient lacks capacity to make medical decisions independently
Apolipoprotein E genotyping was performed and included in study analyses	Clinical apolipoprotein E genotyping is performed prior to initiating treatment to assess an individual’s risk of ARIA
Exclusion Criteria	
Significant neurological disease affecting the central nervous system other than AD, that may affect cognition or ability to complete the study, including but not limited to, other dementias, serious infection of the brain, Parkinson’s disease, multiple concussions	Non-AD neurologic condition that may be significantly contributing to cognitive or behavioral impairment Patients with autosomal dominant AD, if the mutation is associated with high prevalence and burden of cerebral amyloid angiopathy (CAA) Patients with AD due to Down syndrome Patients with stroke or transient ischemic attack within the past 12 months or any history of seizures
Current serious or unstable illnesses including cardiovascular, hepatic, renal, gastroenterologic, respiratory, endocrinologic, neurologic (other than AD), immunologic, or hematologic disease and other conditions that, in the investigator’s opinion, could interfere with the analyses in the study; or has a life expectancy of <24 months.	Medical condition that may significantly contribute to cognitive impairment or interfere with the patients’ ability to participate in treatment or the clinician’s ability to assess the patient
Prior treatment with a passive anti-amyloid immunotherapy <5 half-lives prior to randomization; have received active immunization against A*β* in any other study; have known allergies to donanemab, related compounds, or any components of the formulation	If previously treated with an A*β*-targeting therapy, must allow washout period of >5 half-lives; must demonstrate current biomarker evidence of A*β* plaques, and must not meet other clinical or MRI exclusion criteria Patients with known allergies to donanemab or related compounds are excluded
Clinically important abnormality at screening, as determined by investigator, in physical or neurological examination, vital signs, ECG, or clinical laboratory test results that could be detrimental to the participant, could compromise the study, or show evidence of other etiologies for dementia	Findings on physical or neurological examination, vital signs, or laboratory tests that may significantly contribute to cognitive impairment or interfere with the patients’ ability to participate in treatment or the clinician’s ability to assess the patient
Poor venous access	Physical condition that impairs the ability of the patient to have intravenous infusions
History of cancer within the last 5 years, except for non-metastatic basal and/or squamous cell carcinoma of the skin, in situ cervical cancer, nonprogressive prostate cancer, or other cancers with low risk of recurrence or spread.	Active cancer that interferes with the ability to comply with donanemab treatment
History of clinically significant multiple or severe drug allergies, significant atopy, or severe posttreatment hypersensitivity reactions (including but not limited to erythema multiforme major, linear immunoglobulin A dermatosis, toxic epidermal necrolysis, and/or exfoliative dermatitis).	History of immunologic disease (e.g., systemic lupus erythematosus, rheumatoid arthritis, Crohn’s disease) or current systemic treatment with immunosuppressants, immunoglobulins, or monoclonal antibodies or their derivatives
Sensitivity to ^18^F-florbetapir or ^18^F-flortaucipir or contraindication to PET	Sensitivity or contraindication to amyloid imaging ligands if amyloid PET is required for confirmation of AD
Primary psychiatric diagnosis other than AD if, in the judgment of the investigator, the psychiatric disorder or symptom is likely to confound interpretation of drug effect, affect cognitive assessment, or affect the participant’s ability to complete the study. Participants with history of schizophrenia or other chronic psychosis are excluded. Participants who are actively suicidal and therefore deemed to be at significant risk for suicide. Participants with a history of alcohol or drug use disorder (except tobacco use disorder) within 2 years before the screening visit	Psychiatric disorder, suicidal ideation, or history of alcohol or substance use that interferes with comprehension of the requirements, potential benefit, and potential harms of treatment and are considered by the clinician to render the patient unable to comply with treatment requirements
Contraindications for MRI, including claustrophobia or the presence of contraindicated metal (ferromagnetic) implants/cardiac pacemaker Evidence on screening MRI of significant abnormality that would suggest another potential etiology for progressive dementia or a clinically significant finding that may impact the participant’s ability to safely participate in the study Presence on screening MRI of amyloid-related imaging abnormalities of edema/effusion, more than 4 cerebral micro-hemorrhages, more than 1 area of superficial siderosis, and any intracerebral hemorrhage greater than 1 cm or severe white matter disease	Contraindications for MRI, including claustrophobia or the presence of contraindicated metal (ferromagnetic) implants/cardiac pacemaker Abnormality on baseline MRI suggesting a non-AD cause for progressive cognitive impairment or a clinically significant finding that may impact the participant’s ability to safely participate in the study Presence on baseline MRI of amyloid-related imaging abnormalities of edema/effusion, more than 4 cerebral micro-hemorrhages, any area of superficial siderosis, any intracerebral hemorrhage greater than 1 cm or severe white matter disease Baseline MRI meets criteria for CAA-related inflammation (see Table 3) Baseline MRI shows evidence of territorial infarcts > 1 cm, >2 lacunar infarcts, cerebral contusion, encephalomalacia, brain aneurysms or other vascular malformations, central nervous system infection, and brain tumors, except for small meningiomas or arachnoid cysts Patients with a bleeding disorder that is not under adequate control (including a platelet count <50,000 or international normalized ratio [INR] >1.5 for participants who are not on anticoagulant) Patients on anticoagulants (coumadin, dabigatran, rivaroxaban, apixaban, betrixaban, or heparin) should not receive donanemab; thrombolytics should not be administered to individuals on donanemab

AD - Alzheimer’s disease; ARIA - amyloid-related imaging abnormalities; CSF - cerebrospinal fluid; ECG - electrocardiogram; MCI - mild cognitive impairment; MMSE - Mini-Mental Status Examination; MoCA - Montreal Cognitive Assessment; MRI - magnetic resonance imaging; PET - positron emission tomography; tPA - tissue plasminogen activator.

**Table 3:** MRI criteria for probable cerebral amyloid angiopathy-related inflammation [52].

Probable CAA-ri
Age ≥40 years of age
Presence of ≥1 of the following clinical features: headache, decrease in consciousness, behavioral change, or focal neurological signs and seizures; the presentation is not directly attributable to an acute ICH
MRI shows unifocal or multifocal WMH lesions (cortico-subcortical or deep) that are asymmetric and extend to the immediately subcortical white matter; the asymmetry is not due to past ICH
Presence of ≥1 of the following cortico-subcortical hemorrhagic lesions: cerebral macrobleed, cerebral microbleed, or cortical superficial siderosis Absence of neoplastic, infectious, or other cause

ICH - intracerebral hemorrhage; MRI - magnetic resonance imaging; WMH - white matter hyperintensity.

**Table 4:** Frequencies of ARIA by *APOE* genotype in the donanemab placebo-controlled trials and open-label addendum.

	Placebo *N* = 999	Donanemab PCT *N* = 984	Donanemab OLA *N* = 1047

**Any ARIA**	142/999 (14.2 %)	364/984 (37.0 %)	335/1047 (32.0 %)
**ARIA-E**	19/999 (1.9 %)	240/984 (24.4 %)	207/1047 (19.8 %)
*APOE4* non-carrier	2/282 (0.7 %)	43/291 (14.8 %)	43/391 (11.0 %)
*APOE4* heterozygote	10/538 (1.9 %)	126/522 (24.1 %)	115/535 (21.5 %)
*APOE4* homozygote	6/174 (3.4 %)	70/168 (41.7 %)	48/114 (42.1 %)
**ARIA-H**	130/999 (13.0 %)	308/984 (31.3 %)	285/1047 (27.2 %)
*APOE4* non-carrier	30/282 (10.6 %)	55/291 (18.9 %)	71/391 (18.2 %)
*APOE4* heterozygote	66/538 (12.3 %)	162/522 (31.0 %)	156/535 (29.2 %)
*APOE4* homozygote	34/174 (19.5 %)	90/168 (53.6 %)	57/114 (50.0 %)

PCT - placebo-controlled trials; OLA - open-label addendum; *APOE4* - apolipoprotein E *ε*4 allele.

**Table 5:** Symptoms observed in patients who develop symptomatic ARIA.

• Headache
• Confusion
• Visual changes
• Dizziness
• Nausea
• Gait difficulty
• Serious ARIA
○ Seizures, including status epilepticus
○ Encephalopathy
○ Focal neurological deficits
○ Death

**Table 6:** Rates of ARIA and intracerebral hemorrhage stratified by antithrombotic and anticoagulant use in donanemab placebo-controlled trials.

	Placebo *N* = 999	Donanemab *N* = 984

**ARIA-E**		
No antithrombotic	10/568 (1.8 %)	144/571 (25.2 %)
Aspirin	7/343 (2.0 %)	75/334 (22.5 %)
Non-aspirin antiplatelet	0/40 (0 %)	11/58 (19.0 %)
Anticoagulant	1/105 (1.0 %)	20/98 (20.4 %)
**ARIA-H**		
No antithrombotic	68/568 (12.0 %)	171/571 (29.9 %)
Aspirin	47/343 (13.7 %)	117/334 (35.0 %)
Non-aspirin antiplatelet	8/40 (20.0 %)	16/58 (27.6 %)
Anticoagulant	14/105 (13.3 %)	28/98 (28.6 %)
**Intracerebral hemorrhage >1cm^3^**		
No antithrombotic	2/568 (0.4 %)	2/571 (0.4 %)
Aspirin	0/343 (0 %)	0/334 (0 %)
Non-aspirin antiplatelet	0/40 (0 %)	1/58 (1.7 %)
Anticoagulant	0/105 (0 %)	0/98 (0 %)

**Table 7:** Description of mild, moderate, and severe radiographic ARIA (from the Prescribing Information).

	Radiographic Severity		
ARIA Type	Mild	Moderate	Severe

ARIA-E	FLAIR hyperintensity confined to sulcus and/or cortex/subcortex white matter in one location <5 cm	FLAIR hyperintensity 5 to 10 cm in single greatest dimension, or more than 1 site of involvement, each measuring <10 cm	FLAIR hyperintensity >10 cm with associated gyral swelling and sulcal effacement. One or more separate/independent sites of involvement may be noted.
ARIA-H Microhemorrhage	≤ 4 new incident microhemorrhages	5 to 9 new incident microhemorrhages	10 or more new incident microhemorrhages
ARIA-H Superficial Siderosis	1 focal area of superficial siderosis	2 focal areas of superficial siderosis	> 2 areas of superficial siderosis

**Table 8:** Management of ARIA depending on the severity of symptoms and the severity of the radiographic ARIA-E or ARIA-H on MRI.

Severity of Changes Observed on MRI	Symptom Description			
	No Symptoms	Mild Symptoms	Moderate Symptoms	Severe Symptoms

	None	Discomfort noted; no disruption of daily activity	Discomfort sufficient to reduce or affect normal daily activity	Incapacitating, with inability to work or to perform normal daily activity
ARIA-E on MRI				
Mild	Continue dosing	Suspend dosing	Suspend dosing	Discontinue dosing
Moderate	Suspend dosing	Suspend dosing	Suspend dosing	Discontinue dosing
Severe	Discontinue dosing	Discontinue dosing	Discontinue dosing	Discontinue dosing
ARIA-H on MRI				
Mild	Continue dosing	Suspend dosing	Suspend dosing	Discontinue dosing
Moderate	Suspend dosing	Suspend dosing	Suspend dosing	Discontinue dosing
Severe	Discontinue dosing	Discontinue dosing	Discontinue dosing	Discontinue dosing

**Table 9:** Medical Center resources needed to manage serious or severe ARIA.

• Emergency department with resources to assess suspected or known ARIA
• MRI scanners readily available for unscheduled scanning of symptomatic patients
• Knowledgeable MRI readers proficient in detection and interpretation of ARIA
• Clinicians with experience in the management of cerebral edema or ARIA
• Hospital ward tiered level of monitoring and management
• Intensive care unit availability
• Electroencephalography available to inpatients
• Neurologist with experience in management of seizures and status epilepticus

**Table 10:** Grading of infusion reactions [75,76].

Grade 1	Grade 2	Grade 3	Grade 4	Grade 5

Mild transient reaction; infusion interruption not indicated; intervention not indicated	Infusion interruption but responds promptly to symptomatic treatment (e.g., antihistamines, acetaminophen, NSAIDs, narcotics, i.v. fluids); prophylactic medication indicated for ≤ 24 h	Prolonged recurrence of symptoms following initial improvement; hospitalization may be indicated for clinical sequelae (e.g., poorly controlled hypertension)	Life-threatening consequences; urgent intervention indicated (may require pressor or ventilatory support)	Death

**Table 11:** Clinical resources required for the safe and effective use of donanemab.

• Clinician skilled in the assessment of cognition to identify individuals with mild cognitive impairment or mild dementia due to Alzheimer’s disease
• MRI available for baseline assessment of cerebrovascular pathology and for monitoring of amyloid related imaging abnormalities (ARIA)
• Radiologists, neurologists, or other clinicians expert in the identification and interpretation of cerebrovascular lesions and ARIA
• Capability to perform amyloid positron emission tomography or lumbar puncture to determine the amyloid status of treatment candidates
• Radiologists, nuclear medicine specialists, neurologists, or other specialists skilled in the interpretation of amyloid imaging or CSF biomarker test results
• Expertise in counseling patients about the meaning and implications of *APOE* genotyping
• Expertise in communicating with patients and care partners regarding anticipated benefits, potential harm, and requirements for administration and monitoring while on donanemab.
• Infusion centers with availability for monthly infusion
• Knowledgeable staff at infusion sites capable of recognizing and managing infusion reactions
• Communication channels established between experts interpreting MRIs and clinicians treating patients with donanemab
• Communication channels established between clinicians treating patients with donanemab and the patient and care partner
• Availability of hospital resources including intensive care unit
• Expertise in the management of seizures and status epilepticus for patients with severe or serious ARIA
• Protocol with standard operating procedures for management of serious and severe ARIA
